# The intimate genetics of *Drosophila* fertilization

**DOI:** 10.1098/rsob.150076

**Published:** 2015-08-05

**Authors:** Benjamin Loppin, Raphaëlle Dubruille, Béatrice Horard

**Affiliations:** Laboratoire de Biométrie et Biologie Evolutive, CNRS UMR5558, Université Claude Bernard Lyon 1, Villeurbanne, France

**Keywords:** fertilization, *Drosophila*, pronucleus, sperm, zygote, egg

## Abstract

The union of haploid gametes at fertilization initiates the formation of the diploid zygote in sexually reproducing animals. This founding event of embryogenesis includes several fascinating cellular and nuclear processes, such as sperm–egg cellular interactions, sperm chromatin remodelling, centrosome formation or pronuclear migration. In comparison with other aspects of development, the exploration of animal fertilization at the functional level has remained so far relatively limited, even in classical model organisms. Here, we have reviewed our current knowledge of fertilization in *Drosophila melanogaster*, with a special emphasis on the genes involved in the complex transformation of the fertilizing sperm nucleus into a replicated set of paternal chromosomes.

## 1. Introduction

The vast majority of animals reproduce sexually through the union of two very different haploid gametes. Fertilization includes a variety of specific nuclear and cytoplasmic events, and represents a research field of obvious fundamental interest. Nevertheless, genetic investigations of animal fertilization are relatively under-represented in modern biology, especially when compared with its immediate companion fields, gametogenesis and early embryo development. Historically, the biology of fertilization has largely benefited from a small number of animal models for which eggs are available in relatively large quantities and fertilization can be controlled experimentally. However, these animals, which include marine invertebrates (essentially echinoderms and molluscs), as well as amphibians, were not amenable for genetic experimentations aimed at identifying factors specifically required for the formation of a diploid zygote [1,2].

There are three recognized types of fertilization in animals, which differ by their mechanisms of karyogamy (the mixing of parental chromosomes). Pronuclear fusion—the fusion of nuclear envelopes (NEs) of male and female pronuclei—is often mentioned in textbooks but is in fact essentially known in sea urchins and sea stars. In these echinoderms, fertilization occurs when the female pronucleus has already formed and pronuclear fusion soon follows the apposition of pronuclei [1]. In the more widespread *Ascaris* type of fertilization, first described by van Beneden in 1884 [3], pronuclei do not fuse but remain separated until the onset of the first zygotic mitosis. Then, paternal and maternal chromosomes intermingle on the metaphase plate of the first zygotic mitosis. The *Ascaris* fertilization type is observed in a wide diversity of animals, including mammals [1,2]. Fertilization in insects, and more generally in arthropods, belongs to the third type, called the gonomeric type. In this case, pronuclei appose without fusing their envelopes, as in *Ascaris*, but the parental chromosomes remain separated until the end of the first zygotic mitosis [4].

Gonomery presents a natural advantage for identifying fertilization mutants in *Drosophila*. Indeed, the separation of parental chromosomes implies that any defect specifically affecting one pronucleus does not necessarily prevent the unalduterated one to perform the first zygotic division within its own hemispindle. When this occurs, the embryo is haploid and usually reaches late embryogenesis before arresting its development. Over the past decade, the characterization of a small number of maternal effect or paternal effect mutants inducing gynohaploid embryo development (i.e. embryos that only have maternally derived chromosomes) has brought new insights into the poorly understood process of male pronucleus formation. Additional mutants uniquely affecting sperm activation or other aspects of zygote formation have been identified, but they remain very rare (table 1). In this article, we have reviewed the published literature relevant to the major steps of fertilization in *Drosophila*, from sperm entry to the first zygotic mitosis.

**Table 1.** *Drosophila* mutants affecting fertilization or zygote formation. Mutants are either considered for their paternal (blue) or maternal (green) contribution.

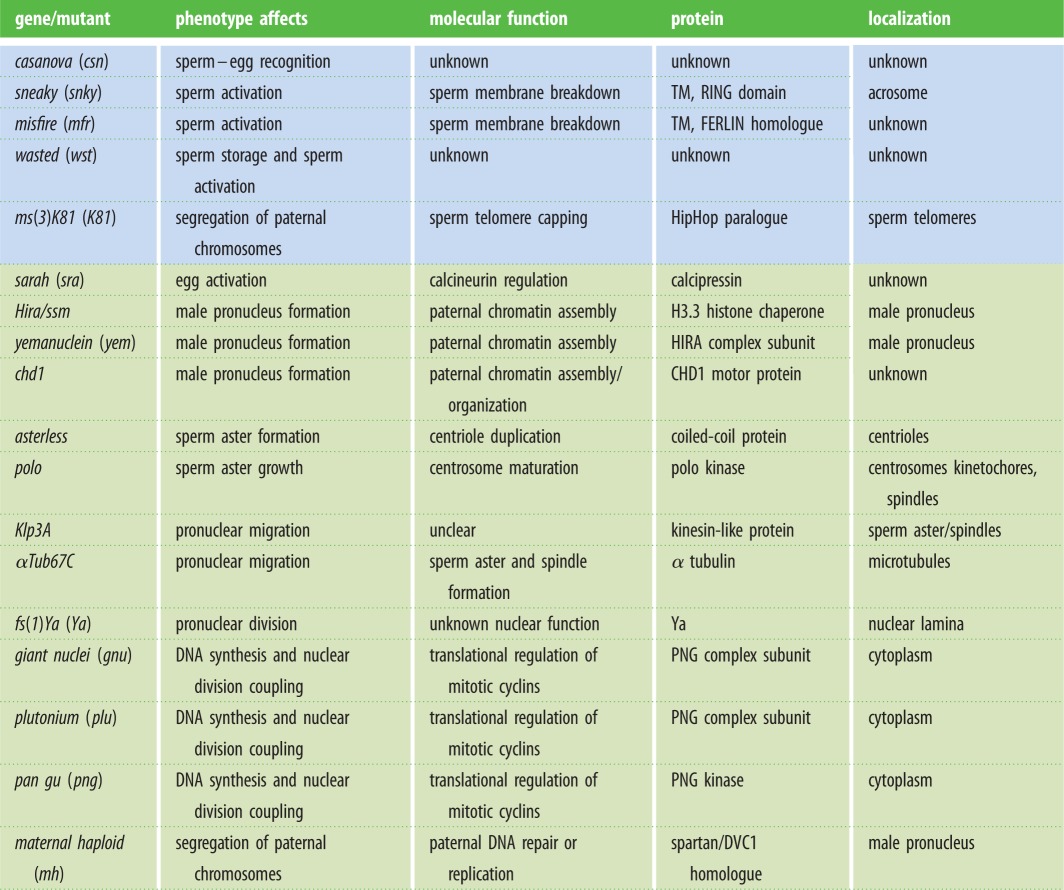

## 2. Generalities about *Drosophila* fertilization

In 1924, Alfred F. Huettner published the first description of the cytological events following egg activation and fertilization in *Drosophila melanogaster* [5]. This seminal work not only included astonishing cytology but also proved to be remarkably accurate and was later used as a foundation for the excellent reviews by Sonnenblick [6] and, more recently, by Foe *et al.* [7].

A major difficulty when observing *Drosophila* fertilization and zygote formation lies in the ultrafast timing of events. The first zygotic division occurs about 15 min after sperm entry [7]. As a matter of comparison, the first cleavage mitosis begins about an hour after fertilization in the parasitoid wasp *Nasonia vitripennis* [8] and 3–4 h in the cricket *Gryllus bimaculatus* [9]. As for other insects, fertilization in *Drosophila* is internal and occurs upon descent of the ovulated oocytes in the uterus. Thus, the earliest events of fertilization, such as sperm activation and paternal chromatin remodelling, are difficult to observe in *Drosophila* as they take place before egg deposition. This limitation is however counterbalanced by the possibility of harvesting freshly laid eggs from many females at a time. Mated females can indeed lay up to 100 eggs per day or, during the egg laying peak, 8 to 10 eggs within a 20-min period [6,10].

*Drosophila* males transfer only a few thousands of gametes during copulation [11]. Mature spermatozoa are stored in seminal vesicles and are transferred to the female genital tract after ejaculation along with a complex mixture of seminal proteins [12]. Mature spermatozoa are released from one of the two sperm-storage organs, the spermatheca and the seminal receptacle (for a recent review on sperm storage, see [13]). Females store 650 sperms, on average, which are used in a highly efficient manner for fertilization [14–16].

**Figure 1.**
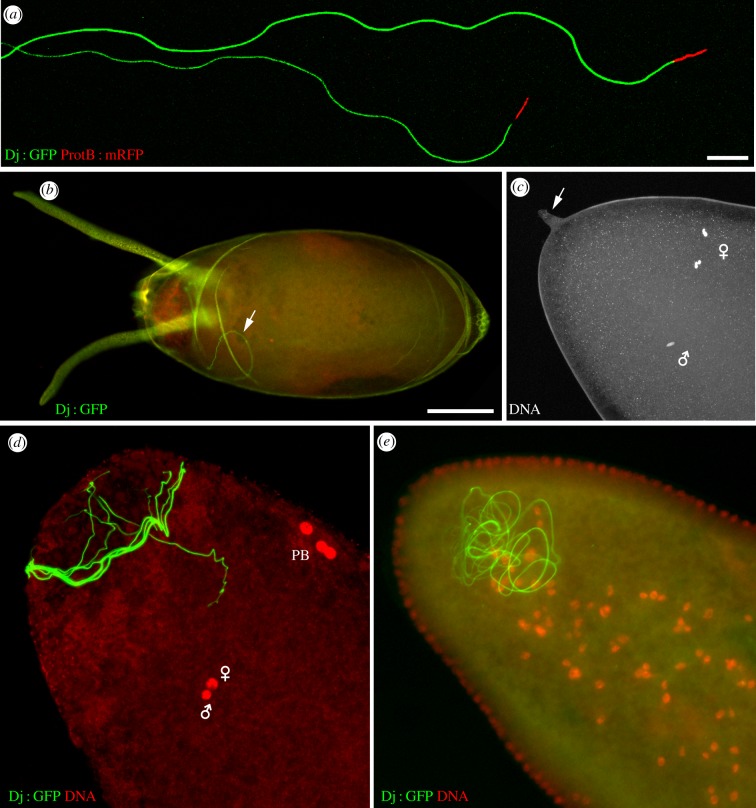
Sperm and fertilization in *D. melanogaster*. (*a*) Isolated *D. melanogaster* spermatozoa from transgenic flies expressing a Don Juan-GFP fusion protein (Dj : GFP) [18] that stains the flagellum and a ProtamineB-RFP fusion protein (ProtB : RFP) [19]. Note that only a fraction of the flagella is visible on this picture. Scale bar, 10 µm. (*b*) In *D. melano*gaster, spermatozoa including the whole flagellum penetrate the egg. A confocal image of a freshly laid egg, with its chorion and dorsal appendages. The flagellum of a Dj : GFP fertilizing spermatozoon is visible in the cytoplasm (arrow). Scale bar, 100 µm. (*c*) A confocal section of a dechorionated egg in metaphase of meiosis II stained for DNA. The vitelline envelope has not been removed and the protruding micropyle is visible at the anterior tip of the egg (arrow). The male pronucleus and female meiotic chromosomes are indicated with symbols. (*d*) An egg at pronuclear apposition stained for DNA (red). The Dj : GFP sperm flagellum (green) is coiled in the anterior region of the egg. PB, polar bodies. (*e*) A blastoderm embryo stained as in (*d*)*.* The flagellum is still detected in the anterior region.

In *D. melanogaster*, the entire 1.8 mm long sperm tail enters the egg cytoplasm through the single micropyle—a specialized opening at the egg surface which allows sperm penetration—and coils within the anterior region of the egg [17] (figure 1). For species with truly gigantic sperm, like *Drosophila hydei* or *Drosophila bifurca* (about 23 and 58 mm, respectively), only a fraction (less than 2 mm) of the flagellum actually enters the egg. For most other species analysed, the entire sperm coils within the anterior region of the egg with species-unique, three-dimensional configurations [20].

Notwithstanding early observations [5], monospermy is the rule in *Drosophila* [21]. Still, approximately 1% of eggs are fertilized by two gametes, and fertilization with multiple spermatozoa can exceptionally occur (up to five spermatozoa observed in a single egg; B.L. 2010, personal observation). Note that polyspermy is otherwise not uncommon in insects [22].

**Figure 2.**
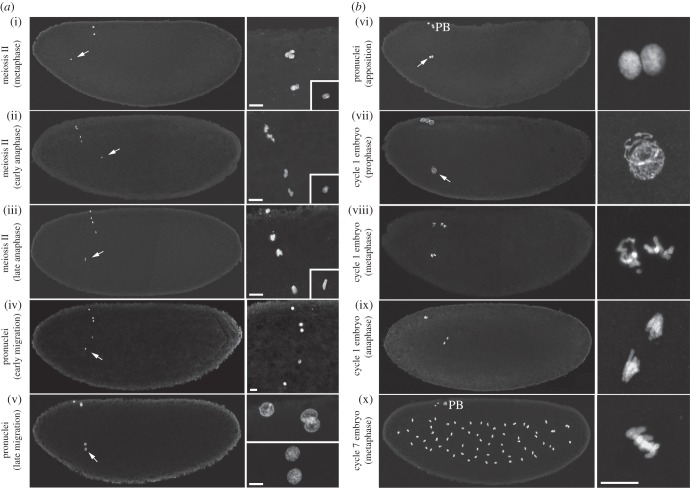
(*a*,*b*) Pronuclear formation and the first zygotic mitosis. (i–x) Confocal images of eggs or embryos at the indicated stages stained for histones to reveal nuclei. Left panels views were reconstituted by fusing two confocal images of the anterior and the posterior regions. Right panels are magnifications of the nuclei (the insets in (i*–*iii) show the male pronucleus). Male pronuclei are indicated (arrows). PB, polar bodies. Scale bars, 10 µm.

**Figure 3.**
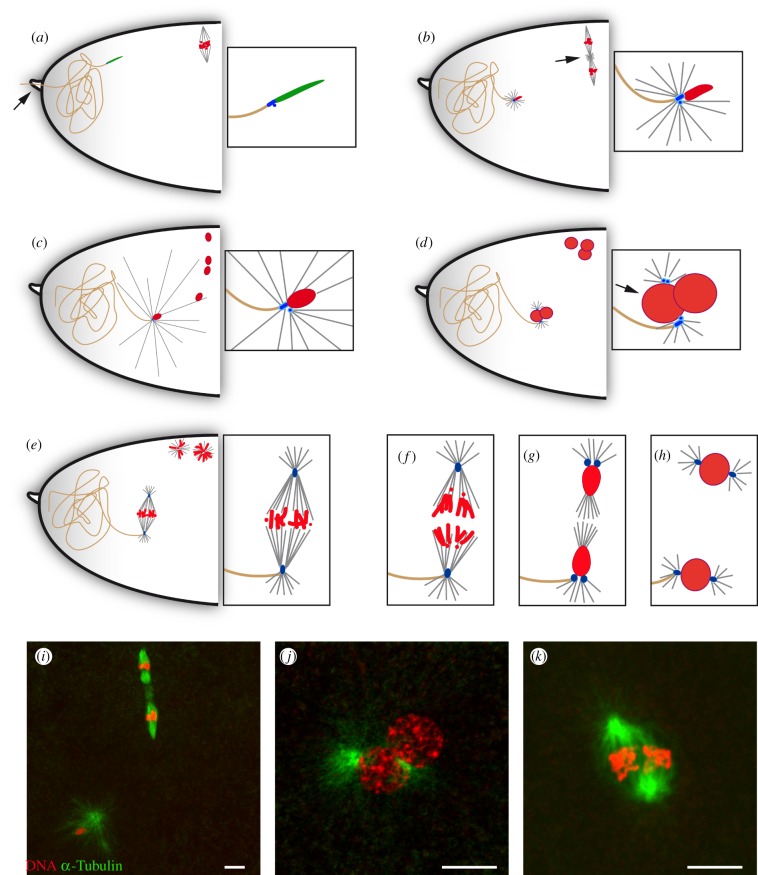
Sperm aster formation, pronuclear migration and organization of the gonomeric spindle. (*a–h*) Schematic of zygote formation in *D. melanogaster*. (*a*) At fertilization, maternal chromosomes are in metaphase of meiosis I. The spermatozoon enters the egg through the micropyle (arrow). The needle-shaped sperm nucleus is still packaged with SNBPs (green) and two centrioles are visible at the junction with the sperm tail: a GC and a centriole precursor, called the PCL (represented in blue). (*b*) Metaphase of meiosis II. The two meiotic spindles are connected by an aster of microtubules, the central spindle pole body (arrow). SNBPs have been replaced by histones and the male pronucleus has begun to decondense. The paternal centrioles recruit PCM and initiate the formation of the sperm aster. (*c*) Pronuclear migration. By the end of female meiosis, the sperm aster has increased considerably in size and captures the innermost female meiotic product, which becomes the female pronucleus. (*d*) Pronuclear apposition. The centrosomes have duplicated and are positioned around the male pronucleus (arrow). All nuclei are in S phase. The three polar body nuclei remain at the egg periphery. (*e*) Metaphase of first mitosis. Each set of parental chromosomes occupies one half of the gonomeric spindle. The polar bodies have condensed into two rosettes of metaphase-like chromosomes (*n* and 2*n*). (*f*) Anaphase of first mitosis. (*g*) Telophase of first mitosis and karyogamy. The centrosomes have duplicated. (*h*) Interphase of second mitosis. (*i–k*) Confocal images of eggs stained for *α*-Tubulin (green) and DNA (red). (*i*) Metaphase of meiosis II. (*j*) Pronuclear apposition. (*k*) Metaphase of first mitosis. Scale bars, 10 µm.

The chromosomes of mature *Drosophila* oocytes are arrested in metaphase of the first meiotic division [23] (for a visual description of *Drosophila* fertilization and zygote formation, see figures 2*a*,*b* and 3). The resumption of female meiosis occurs at egg activation, the process that prepares the egg for the initiation of embryo development. Egg activation also affects maternal pools of mRNAs and proteins, modulates phosphorylation of egg proteins, and modifies the organization of the vitelline membrane and chorion [24,25]. In most animals, fertilization triggers an intracellular calcium signal in the egg that is required for its activation and the initiation of embryogenesis [24,26]. In contrast to vertebrates or marine invertebrates, egg activation in insects is not triggered by fertilization. In unfertilized eggs, meiosis resumes just as it does in fertilized eggs, except that the female pronucleus does not migrate and remains at the egg cortex with polar body nuclei [27]. Interestingly however, two recent studies showed that a calcium wave actually occurs in *Drosophila* mature oocytes as they are ovulated [28,29]. Although the signal that originally triggers this transient rise of intracellular calcium in fly oocytes is not entirely understood, it probably involves a mechanical stimulus associated with ovulation. How the calcium wave regulates downstream effectors of egg activation remains unknown. Interestingly, the Calcipressin Sarah (Sra), which is essential for several aspects of egg activation, including completion of meiosis [30,31], also seems to play a role in calcium wave propagation [29]. In any case, these studies suggest that the existence of a calcium wave at egg activation is a universal feature of animal fertilization.

## 3. Sperm entry and sperm activation

In teleost fishes, cephalopods and insects, eggs have one or several micropyles [32]. In *D. melanogaster*, the unique micropyle encompasses both the chorion and vitelline membrane, and appears as a small, pointed protrusion at the anterior tip of the egg, between the two dorsal appendages (figure 1). *Drosophila* spermatozoa have been shown to move in a tail-leading orientation in the female uterus after insemination [33,34]. However, they enter the micropyle head first after their release from the seminal receptacle, the main sperm-storage organ. In various animal groups, glycosidases and glycosyltransferases are involved in early sperm–egg interaction through their recognition of specific carbohydrates present at the egg surface [35]. In insects, very little is known about the mechanisms controlling micropyle recognition by male gametes. However, two *β*-N-acetylhexosaminidases associated with the sperm plasma membrane could potentially play a role in sperm–egg recognition [36–38]. The *Drosophila* male sterile mutant *casanova* (*csn*) produces sperm that are unable to fertilize eggs [35]. In mutant sperm, *β*-N-acetylhexosaminidase activity is reduced and the enzymatic complex is absent from the plasma membrane overlying the acrosome [36]. Unfortunately, the molecular identity of *csn* is unknown, and the mutant does not map to any of the three known *β*-N-acetylhexosaminidase genes, *Hexo1*, *Hexo2* and *fdl* (Flybase). *Drosophila* sperm membrane also contains an *α*-L-fucosidase expressed in testes [37–39]. In mammals, *α*-L-fucosidases are involved in the binding of sperm heads to the egg *zona pellucida* [40,41]. A functional characterization of the single *Drosophila* α-L-fucosidase gene *Fuca* could thus bring insights into the conservation of these molecules in sperm–egg recognition in insects.

### 3.1. Sperm plasma membrane breakdown

In sperm from marine invertebrates and mammals, the acrosome is a Golgi-derived membranous structure at the apical end of the gamete. At fertilization, exocytosis releases a cocktail of acrosomal enzymes that facilitate penetration of the male gametes through the egg coats and vitelline layer. The exocytic reaction also exposes the inner acrosomal membrane, which eventually fuses with the egg plasma membrane to release the sperm nucleus into the egg cytoplasm [42,43]. In *Drosophila*, the sperm cell penetrates the egg with its plasma membrane, which covers the nucleus and the whole flagellum, indicating that sperm–egg membrane fusion does not occur [44]. Thus, the passage of the male gamete through the micropyle does not involve a typical acrosomal reaction and the associated mechanism remains largely unknown. *Drosophila* spermatozoa nevertheless possess an acrosome at the tip of the nucleus [45], but this membrane-bound structure penetrates the egg at fertilization where it remains detectable throughout zygote formation [46]. Although the *Drosophila* acrosome was described several decades ago [47], its role at fertilization was confirmed only recently through the functional characterization of a conserved, sperm-specific transmembrane protein called Sneaky (Snky) [46]. *snky* was originally identified as one of the very rare paternal effect mutations affecting embryo development (table 1) [48]. In eggs fertilized by *snky* sperm, the sperm nucleus does not decondense, and remains in the egg anterior cortex while maternal chromosomes all gather at the egg periphery and form a tetraploid polar body, as in unfertilized eggs [48]. Fitch and Wakimoto proposed that the *snky* phenotype resulted from a defect in sperm plasma membrane breakdown around the sperm head. Remarkably, and in support of this hypothesis, Wakimoto and co-workers [46] identified Sneaky as a protein that specifically localizes within the membrane overlying the acrosome. Snky belongs to a family of transmembrane proteins with representative members in vertebrates. Although the way Snky could affect sperm plasma membrane integrity remains to be elucidated, its characterization nevertheless implicates the enigmatic fly acrosome in sperm plasma membrane breakdown and sperm activation [46]. In addition, two other male sterile mutants affecting sperm activation have been reported [16,49,50]. The *misfire* (*mfr*) mutants affect the *Drosophila* gene encoding Ferlin [50]. Ferlins are C2 domain-containing transmembrane proteins involved in Ca^2+^ mediated membrane–membrane interactions in various animals and cell types [51]. However, the subcellular distribution of Mfr/Ferlin in *Drosophila* sperm is not yet known and, in contrast to *snky*, *mfr* is also expressed in ovaries where it plays a role in egg patterning [50]. Finally, *wasted* (*wst*) is another mutant that was recently shown to prevent sperm activation [16]. Interestingly, *wst* uniquely affects the control of sperm release from storage organs at ovulation, resulting in rapid loss of sperm stored in the seminal receptacle. Furthermore, *wst* mutant sperm progressively lose their ability to efficiently enter the eggs [16]. The molecular identification of the *wst* gene should help understanding the link between its pre- and post-fertilization phenotypes.

### 3.2. The fate of the sperm flagellum and mitochondrial derivatives

The large sperm tail, which comprises a canonical (9 + 2) microtubule axoneme and mitochondrial derivatives (reviewed in [52]), can be observed long after the initiation of embryo development (figure 1), although it is at least partially degraded. A sperm-derived structure is eventually sequestered in the developing midgut and defecated after larval hatching [53]. Interestingly, Karr & Pitnick [20] observed that the sperm tail is not uniformly degraded in eggs of *Drosophila pachea*, a species with gigantic, helical sperm. Ultrastructural analysis revealed the presence of sperm mitochondrial derivatives in the midgut of hatched *D. pachea* larvae, opening the possibility that this sequestration could participate in the specific elimination of paternal mitochondria [53]. By contrast, recent work in *D. melanogaster* established that paternal mitochondria are actively destroyed after fertilization [54]. Arama and colleagues [54] indeed provided evidence that a network of vesicles common to the endocytic and autophagic pathways disintegrates the sperm plasma membrane over the tail, followed by the mitochondrial derivatives. During this process, which lasts for about an hour, the sperm axoneme is separated from the degraded mitochondrial derivative and persists within the anterior part of the embryo. It thus seems that various strategies are employed in different *Drosophila* species to eliminate paternal mitochondria after fertilization. The role of such a complex elimination process in *D. melanogaster* is however not entirely clear as paternal mitochondrial DNA is destroyed during spermiogenesis, thus ensuring strict maternal inheritance of the mitochondrial genome [55].

## 4. Formation of the male pronucleus

The transformation of a highly compacted and practically inert fertilizing sperm nucleus into a DNA replication-competent male pronucleus is a major event of zygote formation. This unique process of *de novo* chromatin assembly is obviously crucial for paternal chromosome integration in the developing embryo but has rarely been studied *in vivo* at the functional level. *Drosophila* has proved useful in the past decade in providing molecular insights into the conserved chromatin remodelling events, which uniquely occur at fertilization.

### 4.1. Organization of the mature sperm nucleus

The sperm nucleus has the shape of a 9 µm needle and contains highly compacted DNA (figure 1). Sperm DNA compaction is achieved in late stages of spermiogenesis, after the histone-to-protamine transition, which consists in the global replacement of histones with sperm-specific nuclear basic proteins (SNBPs) [45,56]. The histone-to-protamine transition begins with the incorporation of the HMG-box transition proteins Tpl94D (transition protein-like 94D) [57], tHMG-1 and tHMG-2, which are transiently present in canoe spermatid nuclei [58]. These SNBPs are then subsequently and definitively replaced with at least three protamine-like proteins. These include two almost identical paralogous protamine-like proteins (Mst35Ba and Mst35Bb, also known as protamine-A and protamine-B, respectively), which are encoded by two duplicated genes organized in tandem, and Mst77F encoded by a single autosomal gene copy. Mst35Ba/b and Mst77F (146/144 and 215 aa, respectively) are enriched in lysine and arginine residues, but also contain many cysteines that could be involved in the formation of intermolecular disulfide bridges, as in mammalian protamines [56,59]. Interestingly, the Y chromosome contains at least eight additional copies of *Mst77F* (named *Mst77Y*) that potentially encode proteins highly similar to Mst77F [60]. In addition, truncated *Mst35B* copies are present on the Y chromosome but appear non-functional [61]. Unexpectedly, the *Mst35Ba/b* genes are not essential for *Drosophila* male fertility [62,63], suggesting that they could function redundantly with other SNBPs, such as Mst77F or Mst77Y, for the compaction of sperm DNA. In favour of this possibility, Mst77F has been recently shown to efficiently aggregate DNA *in vitro*, suggesting a similar role during spermatid DNA compaction [64]. By the late canoe stage of spermiogenesis, histones are no longer detected, indicating that nucleosomes do not significantly contribute to the organization of sperm chromatin in *Drosophila*. Instead, sperm DNA is uniformly packaged with Mst35Ba/b and Mst77F until fertilization. Notable exceptions are centromeric regions that retain the histone H3 variant Cid in sperm [65]. Cid is actually required to maintain the epigenetic identity of sperm centromeres until fertilization [66], and its transgenerational role is probably conserved in vertebrates that similarly retain the centromeric histone H3 Cenp-A in their gametes [67,68].

### 4.2. Removal of sperm-specific nuclear basic proteins

The removal of SNBPs is the earliest process that probably occurs following sperm entry and activation. In mammals, the formation of intermolecular disulfide bonds between protamines is supposed to contribute to the stability and the compaction of sperm chromatin. At fertilization, these bonds must be reduced to facilitate protamine removal and male nucleus decondensation [69,70]. However, the role of disufide bonds in sperm chromatin compaction remains to be established in *Drosophila*. Additionally, it would also imply the need for a maternal disulfide reductase activity at fertilization, which is yet to be identified. In any case, the eviction of SNBPs from the fertilizing sperm nucleus probably requires dedicated egg proteins. In *Xenopus* eggs, such a role was originally proposed for the conserved and highly abundant protein nucleoplasmin [71]. Nucleosplasmin is a histone chaperone that was identified through its ability to decondense demembranated sperm nuclei *in vitro* [72]. *Xenopus* sperm chromatin is rather unusual, however, as it retains a full load of H3 and H4, while only H2A and H2B are replaced with SNBPs [73]. During early embryo development, nucleoplasmin plays a role in histone storage and release, through the formation of a pentameric structure that could potentially bind five H2A : H2B dimers [74]. In *Drosophila*, the homologous nucleoplasmin-like protein NLP plays a role in the clustering of centromeres [75]. However, a role for NLP in sperm chromatin remodelling at fertilization has not been investigated at the functional level. A recent study reported that NLP, together with its paralogue nucleophosmin, the histone chaperone NAP-1 and a new factor called P32 are capable of removing Mst35Ba/b proteins that were complexed with plasmid DNA *in vitro* [76]. However, clear evidence that these proteins actually mediate SNBP eviction *in vivo* is still missing.

### 4.3. *De novo* assembly of paternal chromatin and nuclear decondensation

**Figure 4.**
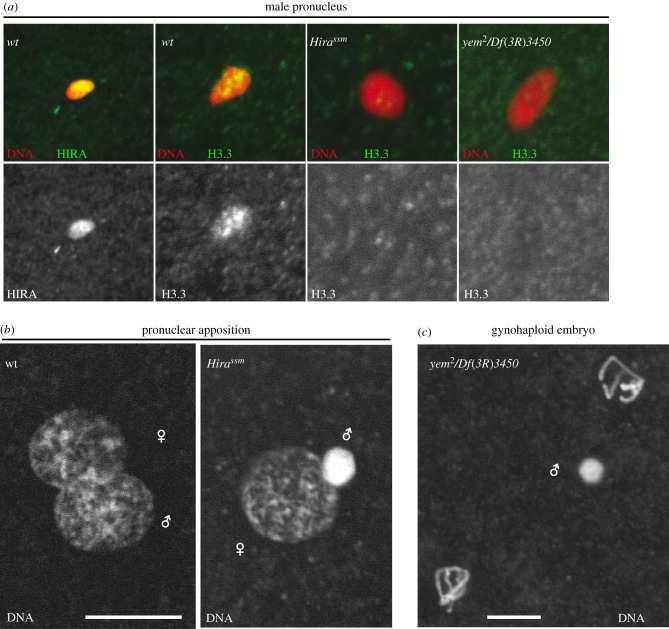
HIRA complex is essential for paternal chromatin assembly at fertilization. (*a*) Confocal images of fertilized eggs in meiosis II stained for DNA (red) and HIRA or histone H3.3 variant (green). In wild-type embryos (wt), the maternal histone H3.3 chaperone HIRA specifically accumulates in the male pronucleus at fertilization, where it promotes *de novo* assembly of nucleosomes independently of DNA synthesis. Newly assembled paternal chromatin is specifically enriched in H3.3. HIRA cooperates with YEM for *de novo* assembly of paternal chromatin after the removal of SNBPs. In eggs laid by *Hira^ssm^* or *yem^2^/Df*(*3R*)*3450* females, H3.3 is not deposited in the male pronucleus. (*b*) Pronuclear apposition in wt and *Hira^ssm^* eggs. The male pronucleus appears abnormally condensed in mutant eggs. (*c*) A gynohaploid embryo laid by a *yem^2^/Df*(*3R*)*3450* female in prophase of the second mitosis. The male pronucleus is visible between the two haploid nuclei. Scale bars, 10 µm.

Genome-wide assembly of nucleosomes on paternal DNA immediately follows the rapid loss of SNBPs from the decondensing male nucleus. This chromatin assembly activity is entirely dependent on maternally provided histones and nucleosome assembly factors, and it occurs well before the onset of the first round of paternal DNA replication. Paternal chromatin assembly at fertilization thus represents a unique case of a genome-wide, replication-independent (RI) nucleosome assembly process [77,78]. Assembly of paternal nucleosomes occurs very rapidly following sperm entry. In eggs observed in metaphase of the second meiotic division, the male nucleus stains for anti-histone antibodies, indicating that its chromatin is already organized into a nucleosome-based configuration (figure 4). The functional characterization of this key event begun with the identification of *sésame* (*ssm*), a maternal effect mutation inducing gynohaploid embryo development [79]. The male nucleus in *ssm* mutant eggs is largely devoid of histones and fails to decondense normally [65]. As a consequence, paternal chromosomes do not replicate and the embryo develops with the sole set of maternally derived chromosomes. *ssm* is a point mutant allele of *Hira*, which encodes a highly conserved histone chaperone characterized by a N-terminal WD40 protein interaction domain [80]. In contrast to the CAF-1 (chromatin assembly factor 1) complex, which allows the assembly of nucleosomes at DNA replication forks, HIRA possesses the remarkable ability to deposit histones in a RI manner [81]. Furthermore, while the CAF-1 complex assembles nucleosomes with canonical histones expressed in S phase, including H3 (also called H3.2), the HIRA-dependent nucleosome assembly pathway specifically uses the highly conserved histone H3 variant H3.3, which is expressed throughout the cell cycle [82]. In *Drosophila*, H3.2 and H3.3 only differ at a small number of critical residues that drive their respective nucleosome assembly pathways [83]. At fertilization in *Drosophila*, HIRA also specifically assembles H3.3 nucleosomes in the male pronucleus, despite the presence of large quantities of both types of H3 in the egg [77,78,80,84]. Consequently, the newly assembled paternal chromatin is almost entirely composed of H3.3-containing nucleosomes, whereas maternal chromosomes are essentially packaged with H3.2-containing nucleosomes [80].

The implication of the HIRA/H3.3 nucleosome assembly pathway in male pronucleus formation is not restricted to *Drosophila*. In mouse and human eggs, where the majority of nucleosomes are replaced with protamines in sperm, H3.3 is also massively incorporated in the male nucleus at fertilization [85–87], and the requirement of HIRA/H3.3 in male pronucleus formation was recently demonstrated in mice [88–90].

In mammals, HIRA functions as a complex that comprises at least two additional subunits, Ubinuclein1 and Cabin1 [82]. *Drosophila* does not seem to possess a Cabin1-related protein [91]. However, Ubinuclein1 is represented by the Yemanuclein (YEM) protein, a DNA-binding protein with a strong expression in the female germline [92]. Like HIRA, YEM is absolutely required for paternal chromatin assembly, and *yem* and *Hira* maternal effect mutant phenotypes are indistinguishable [93] (figure 4). A remarkable property of the HIRA complex lies in its very efficient targeting of the male nucleus within the comparatively gigantic volume of the egg cell. Both HIRA and YEM are present in the male nucleus at the onset of decondensation, concomitantly or immediately following SNBP eviction. The removal of these sperm chromosomal proteins is expected to transiently expose paternal DNA. It is thus tempting to propose that the HIRA complex as a whole, or through the DNA-binding capacity of YEM, could specifically recognize and bind paternal DNA at the protamine-to-histone transition. Interestingly, YEM is actually required for HIRA localization in the male pronucleus [93]. In addition, human HIRA, UBN1 and CABIN1 can all bind DNA *in vitro*, suggesting that the complex uses this property to restore chromatin at nucleosome-depleted regions [94].

Male pronucleus decondensation is also dependent on maternal chromo-helicase-DNA-binding protein 1 (CHD1), a SWI2/SNF2 family of ATP-dependent chromatin remodellers involved in the sliding of nucleosomes along DNA [95]. In *chd1* mutant eggs, paternal chromatin assembly seems to occur, at least partially, but the shape of the improperly decondensed male nucleus appears highly variable [77,95]. The actual function of CHD1 in sperm chromatin remodelling is not clear, and it is currently not known whether this factor localizes to the fertilizing sperm nucleus. The mutant phenotype and its known remodeller activity nevertheless suggest that CHD1 is involved in the regular distribution of newly assembled nucleosomes along paternal chromosomes.

### 4.4. Histone variants and histone marks in the zygote

Although newly assembled paternal chromatin consists almost exclusively of H3.3-containing nucleosomes, this unique enrichment of H3.3 on paternal chromosomes does not seem to play any role *per se* in *Drosophila*. In fact, during the early cleavage divisions, the initial stock of paternal H3.3 nucleosomes is rapidly diluted by the successive waves of replication-coupled assembly of H3.2 nucleosomes [84]. Remarkably, viable, diploid embryos can develop in the absence of H3.3, when the replicative H3.2 histone is maternally provided under the control of the *His3.3B* promoter [96]. This suggests that the HIRA complex can deposit H3.2 onto chromatin in this particular context. It also illustrates the fact that the egg ability to assemble paternal chromatin in a RI manner is crucial, but not the type of histone H3 used during this process. In mouse, however, H3.3 plays additional roles in the zygote, such as establishing paternal pericentric heterochromatin [97]. In contrast to the strict use of the RI histone H3.3 variant, the machinery responsible for *Drosophila* paternal chromatin assembly is less stringent regarding the incorporation of H2A.Z (also known as H2Av), the only other non-H3 core histone variant in flies. Indeed, both H2A-H2B and H2A.Z-H2B dimers are incorporated in the decondensing male nucleus in *Drosophila* [78]. Thus, the assembly of paternal chromatin at fertilization specifically requires the HIRA-dependent deposition of H3.3–H4 tetramers.

In addition to the asymmetric distribution of histone H3.3, several histone post-translational modifications, essentially lysine acetylation and methylation, are differentially distributed on paternal and maternal chromosomes in the zygote. The specific enrichment of histone H4 acetylated on lysines 5 and 12 in the male nucleus [80] simply reflects the massive RI incorporation of newly synthesized histones [98]. For the same reason, acetylated H4 is first detected in the female pronucleus only at the onset of the first S phase. A less expected observation is the complete absence of histone H3 methylation marks in the male pronucleus. This contrasts with the abundance of these marks on post-meiotic maternal chromosomes, including di- and trimethylation of lysines 4, 9, 27 and 36 of histone H3 [80] (B.L. 2009, unpublished data). It is interesting to note, for instance, that the abundance of H3K4me2/3 on maternal chromosomes, which is considered as a mark of active chromatin, is obviously not correlated with gene activity in transcriptionally silent *Drosophila* eggs. Similarly, the presence of the heterochromatin mark H3K9me2/3 on maternal pericentromeric regions is rapidly lost during the early cleavage divisions, questioning the functional signification of this meiotic heritage of H3 methylation marks, which is largely conserved in mammals [85,99,100].

### 4.5. Male pronuclear envelope formation

**Figure 5.**
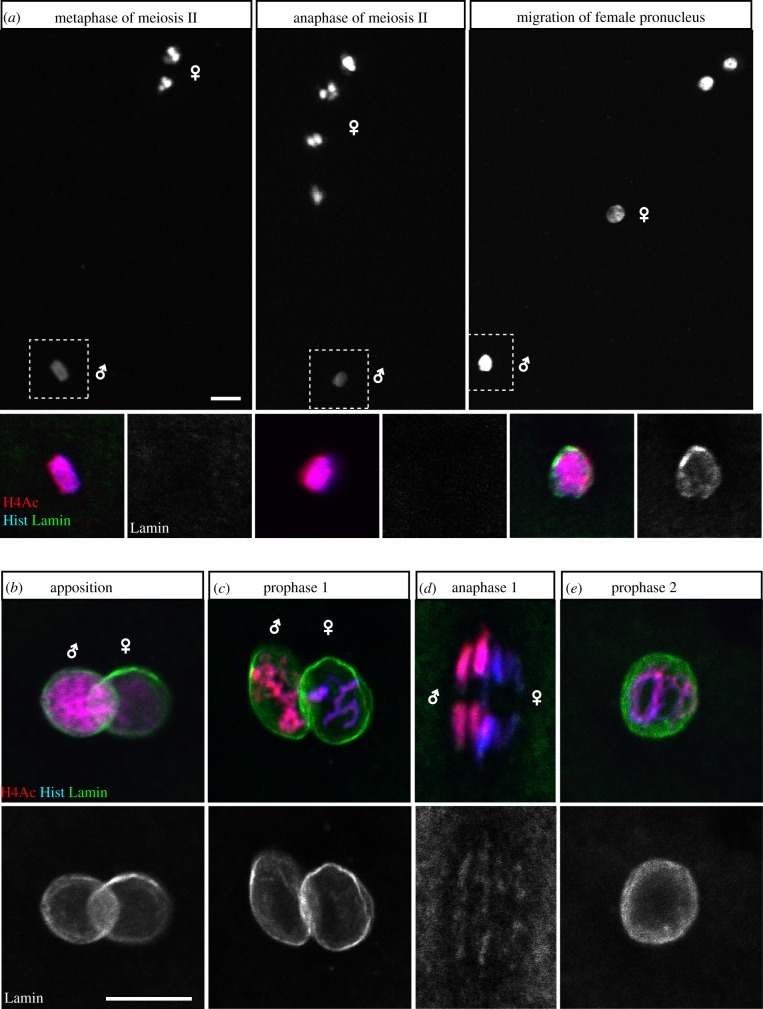
NE dynamics during zygote formation. Confocal images of embryos stained for Lamin Dm0 (green), acetylated histone H4 (red) and histones (blue). (*a*) Top panels show fertilized eggs at the indicated stages. Below are magnifications of the male pronucleus for each egg (insets). At fertilization, Lamin Dm0 is detected for the first time on the male pronucleus at the onset of pronuclear migration. (*b–d*) First zygotic cycle at the indicated stage. Paternal chromosomes are enriched in acetylated histone H4. Male and female pronuclei appose without fusing their NE and divide separately in mitosis 1. In anaphase, Lamin Dm0 is weakly detected on chromosomes. (*e*) Prophase of cycle 2. Scale bars, 10 µm.

The rudimentary sperm NE lacks nuclear pores and is rapidly eliminated at fertilization in most species [2]. In *Drosophila*, the sperm nucleus is also devoid of the major lamina protein Lamin Dm0. After fertilization, Lamin Dm0 is first detected around the male nucleus at the onset of pronuclear migration, indicating that an NE has already formed at this stage [101] (figure 5). The formation of the male pronuclear envelope involves the fusion of egg membrane vesicles at the surface of the male pronucleus, followed by the incorporation of lamins and nuclear pore complexes (NPCs) (reviewed in [102]). However, this process has not been investigated in *Drosophila*. In mouse zygotes, a recent study has established that paternal chromatin assembly is a prerequisite for the incorporation of NPCs at the NE, in a mechanism that depends on the conserved nucleoporin ELYS [88]. Defective NPC incorporation at the envelope would prevent normal swelling of the male pronucleus, as observed in *Hira*-deficient *Drosophila* or mouse eggs [80,88,89].

## 5. Pronuclear migration and apposition

### 5.1. Sperm centrioles and formation of the sperm aster

In *D. melanogaster*, an obligate bisexual species, eggs are acentriolar and sperm centrioles thus represent an essential contribution to the zygote [103]. At fertilization, the recruitment of egg pericentriolar material (PCM) to the sperm-derived centrioles completes the formation of the zygotic centrosomes [104]. The biparental origin of the zygotic centrosome probably explains why unfertilized *D. melanogaster* eggs never develop [105]. Although true parthenogenesis does not exist in *D. melanogaster*, gynogenesis—the development of impaternate diploid progeny from fertilized eggs—occurs at low frequency in the mutant strain *gyn-f9* [106,107]. This strain is homozygous for two uncharacterized autosomal recessive mutations that favour the fusion of the central female meiotic products, allowing the restoration of diploidy. Impaternate progeny are produced when *gyn-f9* eggs are fertilized with sperm from the paternal effect mutant *ms(3)K81* (*K81*), which indeed contributes the required centrioles, but no functional paternal chromosomes (see below). Gynogenesis has also been reported in *yem* mutant females, where the combination of rare non-disjunction of prophase I meiotic chromosomes combined with defective male pronucleus formation led to the exceptional production of viable, impaternate progeny [92,108]. In some *Drosophila* species, however, parthenogenesis (i.e. the development of embryos from unfertilized eggs) is either obligatory, as in *D. mangabeirai* [109], or represents a facultative mode of reproduction, as in *Drosophila* *parthenogenetica* or *Drosophila mercatorum*, for instance [105,110]. In *D. mercatorum*, where parthenogenesis has been investigated in detail, the absence of paternally contributed centrioles in unfertilized eggs is occasionally compensated by *de novo* formation of centrosomes in the egg cytoplasm [111,112], in a way similar to unfertilized eggs from haplodiploid Hymenoptera, which develop into males [113,114]. Interestingly, *de novo* centriole formation can be induced in unfertilized *D. melanogaster* eggs by overexpressing proteins that play a central role in centriole biogenesis, such as Polo-like kinase 4 (Plk4/SAK), Asterless (Asl), DSas-4 or DSas-6 [115–117]. However, *de novo* centriole formation is not sufficient for successful parthenogenesis, which additionally requires the restoration of diploidy and the maturation of centrosomes during embryo development [112].

Although *Drosophila* sperm were originally thought to carry a single, giant centriole (GC) or basal body [118,119], recent work from Avidor-Reiss and co-workers [120,121] demonstrated that spermatozoa also contain a centriole precursor called the proximal centriole-like (PCL), closely associated with the GC. The duplication of the GC and PCL, and the recruitment of maternal PCM to sperm centrioles, allow the formation of the zygotic centrosomes, which remain closely associated with the male nucleus [122]. PCM proteins provided by the egg cytoplasm notably include centrosomin (Cnn), *γ*-tubulin and CP190 [103]. The centrosome derived from the GC remains attached to the sperm flagellum during the embryonic cleavage divisions [123] (figure 3).

The zygotic centrosomes are first required to form the sperm aster, a giant aster of microtubules involved in the migration of the female pronucleus towards its male counterpart (figure 3). The sperm aster enlarges by the end of meiosis II and makes contact with the anterior egg cortex [119,124]. In *asl* mutant eggs, paternal centriole duplication is abolished and pronuclear migration fails [122], as a likely consequence of defective sperm aster formation or function. Sperm aster formation is also compromised in mutants affecting *abnormal spindle* (a*sp*) and *Ran*, whose associated proteins are both involved in microtubule assembly [125,126], as well as in some mutant alleles of the maternal *α-Tubulin at 67C* (*αTub67C*) gene [127,128]. In *asp* and *αTub67C* mutants, a mitotic spindle is formed around the sole set of paternal chromosomes, which can occasionally divide, giving rise to androhaploid embryos.

### 5.2. Migration of the female pronucleus

Female meiosis rapidly resumes at egg activation and, in our experience, eggs have usually reached meiosis II by the time they are collected and fixed for cytological observations. The anastral spindles of the second meiotic division are organized in tandem on an axis that is approximately orthogonal to the egg surface. The spindles are connected with a central microtubule organizing centre, which is positive for γ-tubulin but lacks centrioles. At the end of meiosis, the four meiotic products are aligned in a highly stereotypical manner (figures 2*a* and 3). In unfertilized eggs, the four haploid nuclei decondense, replicate their DNA and eventually gather below the egg surface [27]. They then enter an abortive M phase where the 16 metaphase-like chromosomes usually organize into a tetraploid rosette, with all the centromeres oriented towards the centre of this structure. In fertilized eggs, the innermost meiotic product systematically becomes the female pronucleus and starts its migration towards the more centrally located male pronucleus. In this case, the central meiotic products are generally the first to combine, forming a diploid rosette while the most external one condenses into a haploid rosette. Usually, a single triploid rosette containing the three unused products is eventually formed at the egg periphery and remains arrested in this configuration throughout syncytial embryo development.

Although the spatial position of the female pronucleus obviously favours its capture by the sperm aster, this strict selection probably requires yet unknown additional clues. In addition, the mechanism ensuring the specific interaction of the female pronuclear envelope with the microtubules of the sperm aster is not well understood. Interactions of nuclei with cytoskeletal features are generally mediated by LINC (linker of nucleoskeleton and cytoskeleton) protein complexes [129]. These complexes combine proteins with SUN and KASH domains that localize in the inner and outer nuclear membranes, respectively. Interestingly, LINC complexes have been implicated in the association of the centrosomes with the male pronucleus in *C. elegans* and zebrafish eggs [130,131]. In *Drosophila*, the conserved SUN domain protein Spag4 is a testis-specific protein involved in the attachment of the basal body to the spermatid nucleus during spermiogenesis [132]. *Drosophila* possesses one additional SUN protein (Klaroid) and two KASH domain proteins (Klarsicht and Msp300), but they do not seem to play any role in pronuclear migration [133,134].

The migration of the female pronucleus along the microtubules of the sperm aster must involve a minus-end-oriented, microtubule-associated motor protein. In bovine and primate oocytes, cytoplasmic dynein is indeed required for pronuclear migration, and this motor protein remarkably accumulates at the surface of the female pronucleus but not the male pronucleus [135]. In *Drosophila*, whether cytoplasmic dynein plays any role in this process is not known. Interestingly, female pronuclear migration is actually prevented by mutations in *kinesin-like protein 3A* (*klp3A*) [136]. In *klp3A* mutant eggs, the zygotic spindle, which nevertheless forms around paternal chromosomes, frequently arrests in metaphase, suggesting that KLP3A plays additional roles during mitosis in early embryos [136]. However, although one cannot exclude that KLP3A is a minus-end-oriented motor, it is more likely to function in the opposite direction, like its homologue KIF-4 [137]. In this case, its role in female pronuclear migration would be indirect, perhaps by allowing the normal function of the sperm aster. During mitosis, KLP3A and its partner Feo are involved in the recruitment of the kinase Polo to the spindle midzone [138]. Polo is a conserved regulator of various aspects of cell division, including centrosome maturation, spindle formation and cytokinesis [139]. Interestingly, in *polo^1^* hypomorphic mutant eggs, the sperm aster fails to grow, thus preventing pronuclear migration [124]. It would thus be interesting to clarify the functional relationship between KLP3A and Polo during sperm aster formation and pronuclear migration. Finally, two additional proteins annotated as kinesin-like proteins have been proposed to participate in pronuclear migration: non-claret disjunctional (Ncd) and Subito [127,140]. Although their actual implication in this process remains to be established, the fact that Ncd (and probably Sub) is a minus-end-oriented motor [141] is compatible with such a function.

The swelling of both male and female pronuclei occurs progressively during the migration phase and the female pronucleus usually appears slightly larger than its male counterpart at the time of apposition [119]. The mechanism by which pronuclear envelopes remain in contact is unknown.

## 6. The first zygotic division and karyogamy

### 6.1. Pronuclear DNA replication

Following apposition, both pronuclei (as well as the three polar body nuclei) continue to swell until they reach approximately 10 µm in diameter. The first zygotic round of DNA synthesis probably initiates shortly before apposition, based on the nuclear detection of the replication factors proliferating cell nuclear antigen and DNA polymerase *α* [65,142]. DNA replication then occurs synchronously in apposed pronuclei and polar body nuclei. The onset of the first zygotic cycle marks the transition between the oocyte/egg meiotic divisions and the rapid embryonic nuclear cycles. A few maternal genes appear to be specifically involved in this transition. *Young arrest* (*Ya* or *fs(1)Ya*) encodes a maternal nuclear lamina protein that is detected at the NE of apposed pronuclei and interphasic polar bodies [143,144]. The majority of eggs laid by *Ya* mutant females are arrested at the pronuclear apposition stage, probably at the S to M transition of the first zygotic cycle [143,145]. Although *Ya* mutations affect pronuclei in a unique manner, the presence of Ya at the NE throughout early embryo development suggests that it is required not only for the first zygotic division but for all syncytial mitoses [143]. Furthermore, the pronuclear arrest observed in *Ya* mutant eggs does not simply result from a global disorganization of the lamina, as it does not affect the localization of Lamin Dm0 [146]. This instead suggests that Ya establishes, in a more subtle manner, a nuclear architecture compatible with the unique streamlined nuclear cycles of early *Drosophila* embryos [145].

*giant nuclei* (*gnu*) is the founding member of another class of three maternal effect mutants affecting the egg-to-embryo transition. *gnu*, *plutonium* (*plu*) and *pan gu* (*png*) all affect the coupling of DNA replication with nuclear divisions in eggs and early embryos, resulting in the formation of a small number of extremely large nuclei [147,148]. In the most extreme cases, mutant embryos contain five giant nuclei that correspond to the endoreplicated pronuclei and polar bodies. These mutations all affect a trimeric complex, comprising the PNG kinase and its two regulatory subunits, which is required to promote the translation of cyclin B mRNAs in eggs and early embryos [149]. The uncontrolled succession of S phases in these mutants indistinctly affects the male and female pronuclei as well as the polar bodies. In addition, weaker *png* alleles allow for a limited number of cleavage divisions before the formation of giant nuclei [150]. The control of DNA replication by the activity of the PNG kinase complex is thus not restricted to the first zygotic cycle but is likely to be operating throughout early embryo development (reviewed in [151]).

### 6.2. Completion of the first zygotic mitosis

Chromosome condensation in prophase of the first zygotic mitosis immediately follows the completion of the first S phase. In the gonomeric spindle, both hemispindles are connected at their poles with an aster of microtubules [5–7,119] (figure 2). Maternal chromosomes usually start to condense slightly ahead of paternal chromosomes and are the first to congress on the metaphase plate [119]. The gonomeric nature of the zygotic spindle keeps the parental chromosomes physically separated until telophase, when NEs reform [119]. This is clearly illustrated by immunodetection of Lamin Dm0, which persists around each set of chromosomes until anaphase of the first zygotic mitosis (figure 5). Although paternal and maternal chromosomes normally enter anaphase synchronously, the perturbation of one set of chromosomes does not prevent the segregation of the other one (see below). It is indeed generally admitted that a DNA replication checkpoint is lacking or is not efficient in early *Drosophila* embryos, as suggested by the unperturbed amplification of centrosomes in embryos injected with the DNA replication inhibitor aphidicolin [152]. In addition, the gonomeric spindle seems to lack a checkpoint ensuring the faithful segregation of all chromosomes.

The selective elimination of paternal chromosomes was observed for the first time in cytoplasmic incompatible eggs of the sibling species *Drosophila simulans* [153,154]. Cytoplasmic incompatibility (CI) occurs in a wide diversity of insects when males infected with the endosymbiotic bacteria *Wolbachia* are crossed with uninfected females [155,156]. In *D. simulans*-incompatible eggs, paternal chromosomes appear improperly condensed and lag on the metaphase plate during anaphase of the first zygotic division [153]. Their incapacity to segregate correctly at the first mitosis results in non-viable aneuploid or haploid embryos. CI thus favours the spreading of *Wolbachia* in fly populations through the elimination of uninfected eggs.

Although the molecular mechanism of CI remains a mystery, it probably involves a reversible modification or perturbation of sperm chromatin by *Wolbachia* factors expressed in the male germline. Such a modification, which can be removed or ‘rescued’ at fertilization if the egg is also infected, could impede or delay sperm chromatin remodelling and paternal DNA replication, resulting in abnormal condensation of paternal chromosomes in metaphase [157–159].

*maternal haploid* (*mh*, originally named *fs(1)1182*) is a maternal effect mutation that induces a phenotype very similar to *Wolbachia*-mediated CI [160]. *mh* and a few other mutants (isolated in the same genetic screen and subsequently lost) were the first isolated female sterile mutations producing haploid embryos [7,161]. In *mh* mutant eggs, paternal chromosomes fail to condense properly in metaphase of the first mitosis and form a chromatin bridge during division. Consequently, the majority of *mh* embryos arrest development after a few rounds of aberrant divisions producing aneuploid nuclei, but about 20% of *mh* embryos develop as gynohaploids [160]. Interestingly, *mh* encodes the fly orthologue of Spartan/DVC1, a conserved metalloprotease involved in the regulation of translesion synthesis (TLS) in human cells [142]. TLS is a general DNA damage tolerance mechanism that allows the replication fork to progress across certain types of DNA lesions, such as UV-induced DNA interstrand cross-links, for instance. In mouse, the Spartan knockout is zygotically lethal early in embryogenesis, and is associated with incomplete DNA replication and chromatin bridges in cultured cells [162]. This function is apparently conserved in *Drosophila*, as *mh* mutant larvae are hypersensitive to UV irradiation [142]. However, the specific role of MH in the male pronucleus remains elusive. Nevertheless, its unique and transient accumulation in the male nucleus before the first S phase suggests a role for MH in preparing the uniquely constrained sperm DNA for replication.

Although *mh* is phenotypically unique among gynohaploid maternal effect mutants, a similar phenotype is observed in eggs fertilized by sperm from *ms(3)K81* (*K81*) mutant males [163]. *K81* is one of the rare paternal effect mutants affecting embryo development [48,106,164] (table 1). The defective segregation of paternal sister chromatids derived from *K81*-mutant sperm actually results from a defective telomere capping [165,166]. *K81* indeed encodes a male-germline-specific paralogue of the more general telomere capping protein HipHop [165]. Although defective telomere capping is detrimental in early male germ cells and during meiotic divisions [167], the loss of capping proteins in spermatids of *K81* mutant males does not prevent normal sperm maturation. At fertilization, however, unprotected sperm chromosome ends are recognized as DNA double-strand breaks and ligated by the DNA repair machinery. The formation of dicentric paternal chromosomes presumably occurs during pronuclear formation and invariably results in chromatin bridging at the first mitosis [165,166]. Although apparently similar at the cytological level, *mh* and *K81* phenotypes in fact result from very different defects affecting paternal chromosomes. It is thus likely that more genes specifically required for the integration of paternal chromosome in the zygote remain to be discovered.

## 7. Conclusion

The formation of a diploid zygote concentrates many cellular and molecular events not to be found again in the rest of development or adult life. We present in this article only a partial view of *Drosophila* fertilization, which is largely guided by the still limited number of functional studies that specifically focus on this funding event of embryo development. The genetics of fertilization in *Drosophila* has largely benefited from the characterization of rare mutants inducing haploid embryo development. However, the probability of identifying new mutants of this class from existing collections is slim. Although the design of new forward genetic screens aimed at isolating new mutations is certainly possible, the rarity of these mutants, as well as the considerable effort generally required to identify the corresponding genes, can be discouraging. Fortunately, highly efficient reverse genetic techniques have recently become available and they open new perspectives for the development of this field. The design of an efficient gene knock-down system in the female germline, based on inducible small-hairpin RNAs [168], greatly facilitates the rapid screening of maternal-effect phenotypes for selected genes. It also provides an advantageous alternative to the analysis of germline mutant clones traditionally used to investigate the maternal contribution of genes essential for adult viability. In addition, the rapid development of powerful gene editing technologies based on the CRISPR/Cas9 system [169] allows for an even deeper exploration of the *D. melanogaster* genome in search for genes involved in the formation of the diploid zygote.
